# The Therapeutic Intensity Score as Predictor of Clinical Outcomes after Total and Partial Adrenalectomy for Unilateral Primary Aldosteronism: Results of a Multicentric Series

**DOI:** 10.3390/jcm12030997

**Published:** 2023-01-28

**Authors:** Umberto Anceschi, Marilda Mormando, Rocco Simone Flammia, Cristian Fiori, Orazio Zappalà, Bernardino De Concilio, Aldo Brassetti, Alessandro Carrara, Maria Consiglia Ferriero, Gabriele Tuderti, Leonardo Misuraca, Francesco Prata, Antonio Tufano, Alfredo Maria Bove, Riccardo Mastroianni, Marialuisa Appetecchia, Giuseppe Tirone, Francesco Porpiglia, Antonio Celia, Giuseppe Simone

**Affiliations:** 1 IRCCS “Regina Elena” National Cancer Institute, Department of Urology, Via Elio Chianesi, 00144 Rome, Italy; 2 IRCCS “Regina Elena” National Cancer Institute, Oncologic Endocrinology Unit, Via Elio Chianesi, 00144 Rome, Italy; 3 “La Sapienza” University of Rome, Department of Maternal Infant and Urologic Sciences, Policlinico Umberto I, Viale del Policlinico, 00161 Rome, Italy; 4 AOU San Luigi Gonzaga, Department of Urology, Regione Gonzole, 10043 Orbassano, Italy; 5 APSS, Santa Chiara Regional Hospital, Department of General Surgery, Largo Medaglie d’Oro, 38122 Trento, Italy; 6 ULSS 7 Pedemontana, San Bassiano Hospital, Department of Urology, Via dei Lotti, 36061 Bassano del Grappa, Italy; 7 APSS, Santa Maria del Carmine Hospital, Department of General Surgery, Corso Verona, 38068 Rovereto, Italy

**Keywords:** Conn’s syndrome, primary aldosteronism, partial adrenalectomy, outcomes, PASO, hypertension

## Abstract

Background: To evaluate the ability of therapeutic intensity score (TIS) in predicting the clinical outcomes of partial (PA) and total adrenalectomy (TA) for UPA. Methods: Between 2011 and 2022, a four-center adrenalectomy dataset was queried for “unilateral adrenal mass” and “UPA” (n = 90). Preoperative TIS of each antihypertensive medication were individually calculated and merged to create a single, cumulative variable. Probability of complete clinical, partial, and absent pooled success rates according to TIS were assessed for the overall cohort by Kaplan–Meier. Cox analyses were used to identify predictors of complete clinical and partial/absent success, respectively. For all analyses, a two-sided *p* < 0.05 was considered significant. Results: At a median follow-up of 42 months (IQR 27–54) complete partial, and absent clinical success were observed in 60%, 17.7%, and 22.3%, respectively. On Kaplan–Meier analysis, TIS < 1 predicted higher complete success rates (*p* < 0.001), while TIS ≥ 1 was predictor of either partial and absent clinical success (*p* = 0.008). On multivariable analysis, TIS < 1 (HR 0.25; 95% CI 0.11–0.57; *p* = 0.001) and adenoma size (HR 1.11; 95% CI 1–1.23; *p* = 0.0049) were independent predictors of complete clinical success, while TIS ≥ 1 (HR 2.84; 95% CI 1.32–6.1; *p* = 0.007) was the only independent predictor of absent clinical success. Conclusions: TIS score and adenoma size may help to identify patients who are likely to be at risk of persistent hypertension after surgery.

## 1. Introduction

Primary aldosteronism, or Conn’s syndrome, represents the most common cause of endocrine hypertension (eHTN), with a prevalence ranging from 5% to 15% in the general population [1]. The overproduction of aldosterone is caused by bilateral adrenal hyperplasia or unilateral adenoma (UPA), which can be treated by medical therapy (antihypertensive drugs) and surgical gland removal, respectively [2]. Although most patients experience a clinical benefit after surgery, in major adrenalectomy series, complete clinical success rate (defined as normalization of blood pressure without the need of antihypertensive medication) ranges from 16 to 72% [3,4]. Commonly, adrenalectomy results in the normalization of aldosterone, rather than in the normalization of systolic blood pressure, with approximately 30–40% of patients still requiring medical treatment after surgery [5]. Recent studies suggested a multifactorial etiology for persistent hypertension (pHTN) after adrenalectomy, identifying age, gender, number of preoperative antihypertensive drugs, and high urinary aldosterone levels as main predictors of a complete clinical success [6]. Furthermore, several prediction scores for the resolution of hypertension after adrenalectomy have been developed but, due to their intrinsic complexity and demographic variability, they are rarely adopted in clinical practice [7,8]. Recently, the therapeutic intensity score (TIS) was introduced as a simple summary measure to assess treatment intensity for individual blood pressure control (BPC) [9]. While this metric has been previously used to compare treatment between patients in longitudinal hypertension cohort studies, its role as a predictor of either clinical success or maintenance of BPC after surgery has not been investigated [10]. The present study aimed to test the ability of TIS in predicting the clinical outcomes of patients affected by Conn’s syndrome with a solitary, functioning adrenal mass, treated with either partial adrenalectomy (PA) or total adrenalectomy (TA) on a multicentric series.

## 2. Material and Methods

From 2011 to March 2022, our prospectively-maintained adrenalectomy database was selected for “unilateral primary aldosteronism” (*n* = 90). In this cohort, 61 patients underwent total adrenalectomy (TA), while 29 patients underwent partial adrenalectomy (PA), respectively. All patients had a diagnosis of UPA confirmed on computed tomography (CT) or magnetic resonance imaging (MRI) or adrenal venous sampling (AVS), according to each center’s preference and availability. Diagnosis of UPA was assessed by saline infusion test (*n* = 60 patients, 66.7%), oral sodium loading test (*n* = 17 patients, 18.8%), fludrocortisone suppression test (*n* = 8 patients, 8.9%) and captopril challenge test (*n* = 5 patients, 5.6%), respectively. Thirty-eight patients (42.2%) had UPA diagnosis confirmation by AVS. In adherence with the guidelines of Endocrine Society, confirmatory AVS was not deemed necessary in patients younger than 35 years with spontaneous hypokalemia and unilateral adrenal mass, showing radiological features suggestive for cortical adenoma [1,11]. Alternative causes of adrenal-related eHTN were excluded before enrollment [12,13]. Exclusion criteria were represented by patients with bilateral adrenal masses, malignant disease, adrenal incidentaloma at pathologic evaluation, missing perioperative data or follow-up <18 months. PA indications were restricted to small adrenal tumors (<3 cm) according to surgeon’s discretion. Routine follow-up consisted of an endocrinologic evaluation at 3, 6, and 12 months after surgery, including blood test analysis and blood pressure measurements. Patients were stratified in two groups according to surgical technique (TA = total adrenalectomy; PA = adrenal-sparing technique). Demographic and perioperative data, as well as pathological and follow-up data, were retrieved from the original dataset, while information on preoperative antihypertensive therapy (number, type of drugs and dosage) were gathered from all patients and their TIS scores assessed [9].

TIS scores of each antihypertensive medication were singularly calculated and then computed into a composite TIS score. A cut-off value of 1 was used to outline two coded variables for summative TIS (≥1: high or <1: low).

Preoperative clinical and demographic characteristics, including gender, age, preoperative hemoglobin (Hb), American Society of Anesthesiology (ASA) score, TIS, clinical tumor size and side, and serum potassium, are listed in Table 1. Intraoperative variables are reported in Table 2. We considered median operative time (MOT), % perioperative complications, % perioperative transfusions, median length of hospital stay (LOS), postoperative Hb, median perioperative Hb drop as main indicators of perioperative outcomes. Complications were recorded and graded according to the Clavien–Dindo classification [14]. Functional results were described according to clinical PASO criteria [6].

Primary endpoints of the study were to identify predictors of complete, partial and/or absent clinical success by using univariable and multivariable Cox regression analysis. Descriptive analyses were used. Differences between continuous variables were assessed with the Wilcoxon rank sum test, while Pearson’s χ2 test was used for categorical data.

Clinical complete, partial, and absent success rates according to PASO criteria were assessed for the overall cohort. According to the coded TIS, the probability of clinical complete, partial, and/or absent clinical success was assessed by the Kaplan–Meier method and compared with the log-rank test. Univariable and multivariable Cox regression analyses were used to identify predictors of partial and absent clinical success. For all analyses, a two-sided *p* < 0.05 was considered significant. Statistical analysis was carried out using the Statistical Package for Social Sciences (SPSS) software v.26.0 (IBM Corp, Armonk, NY, USA).

## 3. Results

A total of 90 eligible patients were identified among centers including 61 TA (group A) and 29 PA (group B), respectively (Table 1).

No significative differences were found between groups in terms of demographic variables (all *p* > 0.2) while median tumor size was significantly higher in TA group (4.2 vs. 2.7; *p* = 0.001). In the PA subgroup, the rate of left-sided adrenal masses was significantly higher (75.9%; *p* = 0.001). No significant difference was displayed for preoperative hypertension rate (86.8% vs. 93.1%; *p* = 0.456) and median cumulative TIS score (range 0.25–1) between groups, with 34.5% patients requiring combined medications (*p* = 0.676), respectively. Moreover, preoperative hypokalemia rates were comparable between groups (*p* = 0.184).

Perioperative and pathologic outcomes are listed in Table 2.

With regard to perioperative outcomes, only median LOS was significantly increased in TA cohort (*p* = 0.038). Complications rate was negligible in both groups (TA: 11.4% vs. PA: 10.3; *p* = 0.488). The distribution by Clavien grade was homogenous between series. A major post-operative complication was observed in one patient of TA cohort, requiring intensive care unit (ICU) monitoring for acute myocardial infarction (AMI). The perioperative transfusion rate was comparable between groups (3.2% vs. 3.4%; *p* = 0.967). UPA was confirmed at pathologic evaluation (Capsulated Adenoma = 77.8%; Diffuse Adrenal Hyperplasia = 22.2%). Functional outcomes according to PASO criteria are reported in Table 3.

At a median follow-up of 42 months (IQR 27–54), a complete, partial, and absent clinical success was observed in 54 (60%), 16 (17.7%), and 20 (22.3%) patients, respectively. Hypokalemia rates were comparable between groups (TA 14.8% vs. PA 10.3%). In the TA cohort, four patients required exogenous steroid replacement (6.5%).

**Figure 1:**
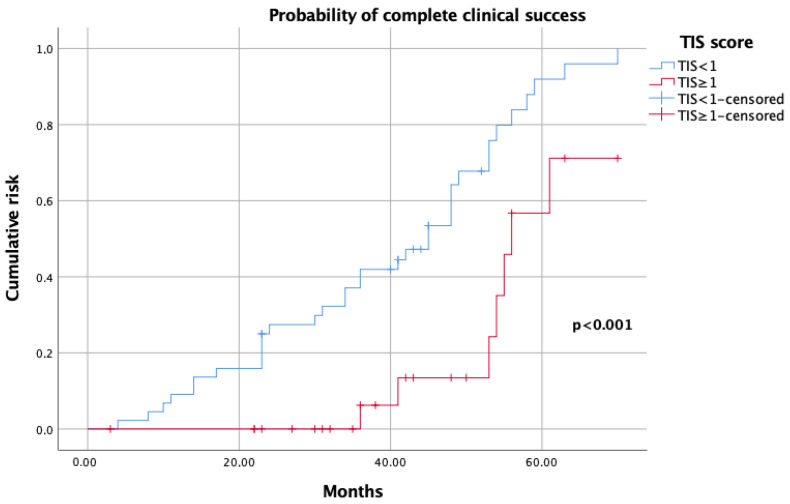
Kaplan–Meier analysis showing probability of complete clinical success, according to TIS score.

**Figure 2:**
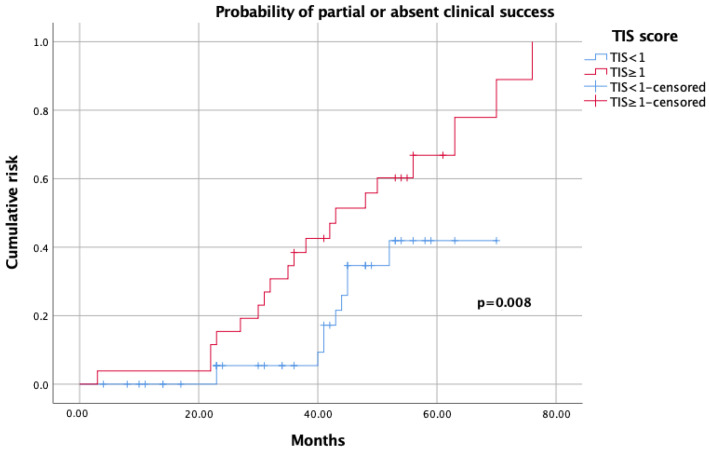
Kaplan–Meier analysis showing probability of partial and absent clinical success, according to TIS score.

On Kaplan–Meier analysis, a TIS score <1 predicted higher complete success rates (*p* < 0.001; Figure 1), while a TIS score ≥1 was a predictor of either partial and absent clinical success (*p* = 0.008; Figure 2).

On multivariable Cox regression analysis, TIS score <1 (HR 0.25; 95% CI 0.11–0.57; *p* = 0.001) and UPA size (HR 1.11; 95%CI 1–1.23; *p* = 0.0049) were significant predictors of complete clinical success (Table 4), while TIS score ≥1 (HR 2.84; 95%CI 1.32–6.1; *p* = 0.007) was the only independent predictor of partial and absent clinical success after surgery (Table 5)

## 4. Discussion

The main goal of surgical therapy for UPA is the removal of the source of aldosterone overproduction, which may theoretically ensure blood pressure normalization [7,15]. Despite the PASO system being recently introduced for standardizing adrenalectomy outcomes, the accuracy of this reporting system remains questionable, since a consistent number of patients (35–66%) affected by UPA may achieve only a partial benefit after surgical treatment, while a clinical failure usually ranges between 0% and 32% [3]. A poor BPC represents an ongoing challenge for clinicians, as there is an established positive association between pHTN and risk of adverse cardiovascular events, such as stroke, myocardial infarction, or heart failure with consequent mortality [16]. Since multiple factors may influence BPC after adrenalectomy, such as age, number of antihypertensive medications, or long-standing PA, several authors attempted to include these in a univocal prediction score [5,7]. Unfortunately, all these clinical tools showed a limited performance, due either to demographic variability or heterogeneity of systolic blood pressure endpoint considered [17,18]. As BPC is affected by continuous variations that occur over a lifetime, the capability of any of these scores to predict the failure or clinical success after adrenalectomy still remains far from being a certainty [5,18]. Furthermore, the use of percentages of defined daily dose (DDD), instead of the number of antihypertensive medications in PASO criteria, did not obviate the issues of adherence to multi-drug therapy or the insufficient lowering effect of a single daily dose, enhancing the risk of pHTN after surgery [19,20,21,22].

TIS score was initially conceived as a tool for estimating the impact of therapeutic modulation for BPC in longitudinal hypertension trials. More recently, it has been identified as a marker of drug titration and compliance in patients failing to achieve BPC [10,23]. Given such a premise, we attempted to evaluate the TIS algorithm as a summary measure that may predict the impact of a single individual antihypertensive dose or concurrent multiple medications on BPC after adrenalectomy. Our study showed interesting findings. On Kaplan–Meier analysis, by using a coded definition for summative TIS, the computation of the relationship between TIS and clinical success showed an inverse probability (*p* < 0.001; Figure 1), while incremental dosing in each drug, rather than the overall number of medications, was associated with a stable and worse response on BPC after adrenalectomy (*p* = 0.008; Figure 2). After controlling for gender, age and surgical approach, on univariable analysis, perioperative complications (any Clavien grade 2–5) and TIS score ≥1 were significantly associated with partial and absent clinical success (all *p* < 0.03), while adenoma size and TIS score <1 were associated with complete clinical success (all *p* < 0.04). On multivariable analysis, a TIS score ≥1 was the only independent predictor of partial and absent clinical success after surgery (*p* = 0.007). Since previous studies showed an association between adenoma size and blood pressure normalization after adrenal gland removal, we may hypothesize that small adenomas are characterized by higher aldosterone levels and, consequently an increased BPC; however, we could not estimate the impact of biochemical variables (such as aldosterone/renin ratios, cortisol values), histochemical (aldosterone synthase), and surgical factors were not included in our dataset [24]. Moreover, age, gender, and BMI, which are usually important factors in considering the benefit and potential risk of adrenalectomy for UPA, did not reach significance in our model, compared to previously published multicentric series [4,6]. The lack of predominance of females as the unusual distribution of median adenoma size observed in our cohort may represent either a reflection of the small series considered or the result of a different contribution rate between referral and low-volume centers included in our dataset. Additionally, as lower cure rates could be expected in older patients, due to either pHTN or unfavorable comorbidities such as hyperkalemia and renal failure, the younger median age of patients considered in our analysis may explain the high rates of clinical success achieved [25].

We firmly believe that an ideal reporting system for UPA should identify patients that may expect an improved BPC or, conversely, an increased antihypertensive dosage after surgery. Nonetheless, according to our results, TIS and adenoma size represent easy clinical predictors, through which physicians can better assess their antihypertensive dosing practices, while urologists may better estimate the expected treatment effects before surgical removal of the adrenal gland.

We acknowledge that this study is not devoid of limitations; therefore, it should be taken as hypothesis-generating. Firstly, the retrospective nature of our series represents an intrinsic bias. The high rate of large adenoma size observed in our series, compared to previously published series, may represent a limitation. Moreover, as cumulative TIS values could represent multiple drugs and combinations, we were unable to provide insight for comparable TIS scores into the real superiority of a treatment (lower doses of combined drugs vs higher single drug dose). Although a single medication may be preferred for supporting patient compliance, a recent study suggested a comparable BPC for multiple drug classes at standard doses, with increased efficacy and reduced adverse effects achieved using a combination of low-dose drug treatments [26,27]. Furthermore, the lack of data regarding immunohistochemical evaluation and somatic mutations on adenoma specimens may have undoubtedly decreased the predictive accuracy of TIS [16,28,29,30].

Despite these limitations, the TIS metric holds promise for its potential introduction in a UPA setting, providing a step forward in estimating and predicting the effect of different antihypertensive regimens on adrenalectomy outcomes. This novel clinical tool may help physicians to inform patients of their expected surgical outcomes and to identify those patients who are prone to a clinical failure and will require a closer follow-up.

## 5. Conclusions

TIS score and adenoma size represent independent prognostic factors to assess the likelihood of a clinical cure versus a long-term antihypertensive medication use. Our findings suggest the introduction of a new algorithm aimed at tailoring adrenalectomy outcomes and their relationship to BPC and quality of care in current clinical practice.

## Figures and Tables

**Table 1:** Baseline and preoperative data.

Variable	Overall Cohort	Total Adrenalectomy	Partial Adrenalectomy	*p*
Age at surgery (median, IQR)	54 (44–65)	54 (44.5–63)	57 (43.5–67.5)	0.408
Follow-up (months, median, range)	42 (27–54)	41 (24–50)	46 (32.7–57.5)	0.223
Gender (*n*, %)				0.519
Male	36 (40%)	23 (37.7%)	13 (44.8%)	
Female	54 (60%)	38 (62.3%)	16 (55.2%)	
ASA score (*n*, %)				0.763
1–2	73 (81.1%)	50 (82%)	23 (79.3%)	
3–4	17 18.9%	11 (18%)	6 (20.7%)	
Adrenal mass size (cm, *n*, IQR)	3 (2–5)	4.2 (2.35–6)	2.7 (1.8–2.85)	**0.001**
Side (*n*, %)				**0.001**
Left	45 (50%)	23 (37.7%)	22 (75.9%)	
Rigth	45 (50%)	38 (62.3%)	7 (24.1%)	
Preoperative Hypertension (*n*, %)				0.456
Yes	80 (88,8%)	3 (86.8%)	27 (93,1%)	
No	10 (11,2%)	8 (13.2%)	2 (6,9 %)	
Preoperative Hypokalemia (*n*, %)				0.184
Yes	63 (70%)	21 (65.6%)	6 (20.7%)	
No	27 (30%)	40 (34.4%)	23 (79.3%)	
Number of drugs (*n*, %)				0.676
One class medication	9 (10%)	7 (11.4%)	2 (6.8%)	
No drugs	50 (55.5%)	32 (52.4%)	18 (62%)	
Combined class medication (≥2)	31 (34.5%)	22 (36%)	9 (31.2%)	
Preoperative TIS score (median, IQR)	0.5 (0.25–1)	0.5 (0.25–1.09)	0.5 (0.25–1)	0.989

**Table 2:** Perioperative and pathologic outcomes.

Variable	Overall Cohort	Total Adrenalectomy	Partial Adrenalectomy	*p*
Preoperative Hb (g/dL, median, IQR)	13.8 (12.8–14.6)	13.4 (12.5–14.3)	14.3 (13.4–14.9)	0.058
Postoperative Hb (g/dL, median, IQR)	12.6 (11.7–13.5)	12.3 (11.6–13.4)	13.3 (11.7–13.5)	0.271
ΔHb (g/dL, median, IQR)	1.1 (0.3–2.1)	1.1 (0.1–1.8)	1.1 (0.4–2.35)	0.337
LOS (days, median, IQR)	4 (3–5)	4 (3–5)	3 (2.5–4)	**0.038**
Overall complications (*n*, %)	10 (11.1%)	7 (11.5%)	3 (10.3%)	0.873
Perioperative transfusions rate (*n*, %)	3 (3.4%)	2 (3.2%)	1 (3.4%)	0.967
Clavien Grade (*n*, %)				
I	*n* = 6	*n* = 4	*n* = 2	
II	*n* = 3	*n* = 2	*n* = 1	0.940
III	-	-	-	
IV	*n* = 1	*n* = 1	-	0.488
V	-	-	-	
Follow-up (months, median range)	42 (27–54)	41 (24–50)	46 (32.7–57.5)	0.223
Histology (*n*, %)				0.209
Adenoma	70 (77.8%)	48 (78.7%)	22 (75.8%)	
Hyperplasia	20 (22.2%)	13 (21.3%)	7 (24.1%)	

**Table 3:** Functional Outcomes according to PASO criteria.

Variable	Overall Cohort	Total Adrenalectomy	Partial Adrenalectomy	*p*
Complete clinical success				
- No medication/Controlled BP	54 (60%)	33 (54%)	21 (72.4%)	0.097
Partial clinical success	16 (17.7%)	14 (23%)	2 (6.8%)	0.136
- Drug Escalation (Controlled BP)	8 (8.9%)	7 (11.5%)	1 (3.4%)	
- Switch to a lower class of medication (Controlled BP)	2 (2.2%)	2 (3.3%)	-	
- No drugs (Moderate BP Reduction)	4 (4.4%)	4 (6.6%)	-	
- Switch to comparable medication (Moderate BP Reduction)	2 (2.2%)	1 (1.6%)	1 (3.4%)	
Absent clinical success	20 (22.3%)	14 (23%)	6 (20.7%)	0.136
- Unchanged dosage medication	14 (15.6%)	9 (14.8%)	5 (17.2%)	
- Increased dosage	3 (3.3%)	3 (4.9%)	-	
- Switch to a stronger class of medication	3 (3.3%)	2 (3.3%)	1 (3.4%)	
Hypokalemia (*n*, %)	12 (13.3%)	9 (14.8%)	3 (10.3%)	0.565

**Table 4:** Univariable and multivariable Cox regression analysis to identify predictors of complete clinical success.

Variable	Univariable Analysis				Multivariable Analysis			
	HR	95.0% CI			HR	95.0% CI		
		Lower	Higher	*p* Value		Lower	Higher	*p* Value
**Age**	0.98	0.95	1.01	0.17	-	-	-	-
**Gender**	1.18	0.62	2.26	0.59	-	-	-	-
**ASA score** **(1–2 vs. 3–4)**	0.65	0.30	1.38	0.26	-	-	-	-
**Adenoma size**	**1.12**	**1.01**	**1.24**	**0.035**	**1.11**	**1**	**1.23**	**0.049**
**Partial vs. Total Adrenalectomy**	1.21	0.65	2.25	0.55	-	-	-	-
**TIS score <1**	**0.25**	**0.11**	**0.56**	**0.001**	**0.25**	**0.11**	**0.57**	**0.001**
**Surgical complications (Clavien 2–5)**	2.76	0.05	7.23	0.77	-	-	-	-

**Table 5:** Univariable and multivariable Cox regression analysis to identify predictors of partial and absent clinical success.

Variable	Univariable Analysis				Multivariable Analysis			
	HR	95.0% CI			HR	95.0% CI		
		Lower	Higher	*p* Value		Lower	Higher	*p* Value
**Age**	1.01	0.97	1.04	0.62	-	-	-	-
**Gender**	1.15	0.54	2.47	0.70	-	-	-	-
**ASA score** **(1–2 vs. 3–4)**	0.85	0.36	1.96	0.70	-	-	-	-
**Adenoma size**	1.13	0.99	1.28	0.06	-	-	-	-
**Partial vs. Total Adrenalectomy**	1.66	0.75	3.66	0.21	-	-	-	-
**TIS score ≥1**	**2.97**	**1.39**	**6.33**	**0.005**	**2.84**	**1.32**	**6.1**	**0.007**
**Surgical complications (Clavien 2–5)**	10.75	1.29	89.4	0.028	6.46	0.76	54.6	0.08

## Data Availability

Not applicable.
